# Flexibility underlies differences in mitochondrial respiratory performance between migratory and non-migratory White-crowned Sparrows (*Zonotrichia leucophrys*)

**DOI:** 10.1038/s41598-024-59715-y

**Published:** 2024-04-24

**Authors:** Emma M. Rhodes, Kang Nian Yap, Paulo H. C. Mesquita, Hailey A. Parry, Andreas N. Kavazis, Jesse S. Krause, Geoffrey E. Hill, Wendy R. Hood

**Affiliations:** 1 Department of Biological Sciences, Auburn University, Auburn, USA; 2 Present Address: Department of Biology, Norwegian University of Science and Technology, Trondheim, Norway; 3 School of Kinesiology, Auburn University, Auburn, USA; 4 Present Address: National Heart, Lung and Blood Institute, National Institutes of Health, Bethesda, USA; 5 Department of Biology, University of Nevada, Reno, USA; 6 Present Address: Aging and Metabolism Research Program, Oklahoma Medical Research Foundation, Oklahoma City, USA

**Keywords:** Ecology, Animal migration, Ecophysiology, Physiology, Respiration

## Abstract

Migration is one of the most energy-demanding behaviors observed in birds. Mitochondria are the primary source of energy used to support these long-distance movements, yet how mitochondria meet the energetic demands of migration is scarcely studied. We quantified changes in mitochondrial respiratory performance in the White-crowned Sparrow (*Zonotrichia leucophrys*), which has a migratory and non-migratory subspecies. We hypothesized that the long-distance migratory Gambel’s subspecies (*Z. l. gambelii*) would show higher mitochondrial respiratory performance compared to the non-migratory Nuttall’s subspecies (*Z. l. nuttalli*). We sampled Gambel’s individuals during spring pre-migration, active fall migration, and a period with no migration or breeding (winter). We sampled Nuttall’s individuals during periods coinciding with fall migration and the winter period of Gambel’s annual cycle. Overall, Gambel’s individuals had higher citrate synthase, a proxy for mitochondrial volume, than Nuttall’s individuals. This was most pronounced prior to and during migration. We found that both OXPHOS capacity (state 3) and basal respiration (state 4) of mitochondria exhibit high seasonal flexibility within Gambel’s individuals, with values highest during active migration. These values in Nuttall’s individuals were most similar to Gambel’s individuals in winter. Our observations indicate that seasonal changes in mitochondrial respiration play a vital role in migration energetics.

## Introduction

For all but a few species of birds, flight is integral to survival. Birds use flight not only to support daily movements, but many species also utilize flight to support biannual large-scale movements across the surface of the globe. Birds preferentially fly rather than locomote by other means because it is their most efficient means of travel; however, flapping flight requires an enormous output of instantaneous energy^1,2^. While many physiological processes that support the sustained flapping flight required for seasonal migration have been studied, quantifying the performance of the key organelles that underlie adenosine triphosphate (ATP) production has been methodologically challenging and, thus, has received limited attention.

There is strong evidence that the adaptations that support the energetic demands of avian migration span from whole organism to cellular processes and include an array of fixed^3,4^ and flexible traits^5–7^. Understanding if avian migrants support greater mitochondrial respiratory performance than non-migrants and if those differences are fixed or flexible is a valuable step in understanding the evolution of this fascinating trait. There is growing evidence from several taxonomic groups that the energetic capacity of a population evolves through changes in the ability of mitochondria to use nutritional substrates and oxygen to make ATP. The adaptations that support these changes may be associated with either fixed or flexible phenotypes, with fixed traits being persistent throughout the year and flexible traits being rapidly reversible^8,9^. For example, the Bar-headed Goose (*Anser indicus*) has the capacity to fly over the Himalayas at a greater altitude than used by other waterfowl species. Their capacity to do so appears to be associated with a single point mutation in the gene coding for cytochrome *c* oxidase (complex IV). This mutation, which is not found in any other waterfowl species, changes oxygen affinity in Complex IV in a fixed manner, altering electron handling and oxygen utilization^10^. In contrast, changes in mitochondrial performance that are associated with hibernation in the thirteen-lined ground squirrels (*Ictidomys tridecemlineatus*) are flexible; liver mitochondria display a nearly complete down- and up-regulation of oxidative phosphorylation as the squirrels’ cycle between hibernation and interbout euthermia^11^. This capacity appears to be attributable, at least in part, to the reversible phosphorylation of key binding sites on complexes I and II^12^.

Birds display a higher capacity to carry oxygen to the cells^13–15^ and more efficient nutritional substrate delivery to tissues, such as flight muscles, prior to and during migration than outside of the migratory period^16–18^. The theory of symmorphosis predicts that the capacity of all parts of a physiological system must be matched in overall functional demand^19^. Thus, it is probable that mitochondria, the organelles that utilize oxygen and substrates to produce ATP, also have adaptations to increase energy demand that match the changes observed at the macro-level. Evidence that mitochondria adapt to the demands of migration include increased citrate synthase (a component of the citric acid cycle and commonly used proxy for mitochondrial volume^20^) and carnitine palmitoyl-transferase as both have been demonstrated to increase in pectoralis during active migration^17,21^. Toews et al.^22^ compared mitochondrial respiration in pectoralis among subspecies of Yellow-rumped Warblers (*Setophaga coronata*) that have two different mitochondrial (mt) genotypes, one migratory and one non-migratory. They found that the migratory mt genotype had a higher oxidative coupling rate (using the acceptor control ratio as a proxy) than the non-migratory mt genotype, suggesting a possible fixed difference between groups^22^. However, this study was limited to only the breeding season.

Demand for ATP drives an increase in oxygen utilization by the electron transport system (ETS) during periods of energy-demanding activity relative to a resting condition^23^. Thus, with numerous adaptations that increase the capacity for oxygen and substrate delivery to the ETS of pectoralis mitochondria during migration, we hypothesized that mitochondrial respiratory performance (as indicated by OXPHOS capacity) would be greater in long-distance migrants than non-migrants. To test this hypothesis, we focused on two subspecies of White-crowned Sparrows, the migratory Gambel’s White-crowned Sparrow (*Zonotrichia leucophrys gambelii*, hereafter Gambel’s) and the non-migratory Nuttall’s White-crowned Sparrow (*Z. l. nuttalli*, hereafter Nuttall’s). Recent studies have revealed that these two subspecies have unique mitochondrial haplotypes^24^, suggesting the possibility that fixed differences in mitochondrial respiratory function are matched to differences in migratory behavior. The White-crowned Sparrow is one of the best-studied birds in North America^25^, providing a strong background for this and future studies.

To test whether individuals in the Gambel’s population possess a high capacity to deliver oxygen and energy to the mitochondria, as is observed in other migrants, we measured hematocrit and hemoglobin. We used hematocrit as a proxy for oxygen-carrying capacity, β-hydroxybutyrate as a measure of ketone bodies supplied to flight muscles, which appears to be an important replacement for glucose while fasting^26^, and citrate synthase as a proxy for mitochondrial volume. Because hematocrit and hemoglobin have been shown to be persistently different between migrants and non-migrants, while migrants display within-population flexibility between the migratory and non-migratory period^3,13^, we predicted that hematocrit, hemoglobin, and citrate synthase would be higher in Gambel’s than Nuttall’s White-crowned Sparrows. We also predicted that these variables would be higher in Gambel’s individuals prior to migration and remain high during migration but that they would be lower outside of the migratory period. In contrast, we predicted that these variables would be consistent throughout the year in Nuttall’s individuals and similar to Gambel’s individuals during the non-migratory period. β-hydroxybutyrate is known to increase in response to the intense demands of migratory flight^26^, and thus, this ketone body is expected to be high only during migration in Gambel’s individuals.

To support the energy demands of migration, Gambel’s individuals were predicted to show elevated OXPHOS capacity and mitochondrial respiratory control during the migratory period compared to the non-migratory period (the OXPHOS flexibility hypothesis). Furthermore, we predicted that if changes in response to a migratory strategy were flexible, then the OXPHOS capacity in Gambel’s individuals would match that of Nuttall’s individuals in non-migratory periods. Alternatively, if the differences between Gambel’s and Nuttall’s in response to different migratory strategies were fixed, we predicted higher OXPHOS capacity and mitochondrial respiratory control in Gambel’s versus Nuttall’s throughout the year (the fixed OXPHOS hypothesis).

## Materials and methods

### Study area and sampling design

Collection took place in 2021; All experimental study was approved by Auburn University Institutional Animal Care and Use Committee (IACUC) (PRN #2019-3549). All methods were performed in accordance with relevant guidelines and regulations. This study compiles with ARRIVE guidelines^27^. We collected two subspecies of the White-crowned Sparrow, Nuttall’s White-crowned Sparrow and Gambel’s White-crowned Sparrow (Fig. S1). Birds were collected at three locations in California: Davis, Yolo County (38.527943, − 121.79061), Inyo National Forest (37.917256, − 119.254190), and Marin Headlands (37.832165, − 122.538683). Birds were captured using baited traps and mist nets. We collected Gambel’s individuals preparing to depart for their spring (vernal) migration in April at Davis (n = 24), during fall migration in September at Inyo National Forest (n = 20)—a site where Gambel’s individuals neither breed nor winter and thus where all captured individuals can be assumed to be engaged in migration—and at the Davis site in December (n = 26), which is well past the end of fall migration and well before the beginning of spring migration^13,28^. Nuttall’s individuals were collected at fall and winter timepoints, coinciding with the collection of Gambel’s individuals. We collected Nuttall’s individuals in September (n = 23) and in December (n = 22) at Marin Headlands. We did not collect this subspecies in April because they would have been breeding. This resulted in a total of five groups, three for Gambel’s and two for Nuttall’s.

### Data collection

Birds were trapped throughout the day from sunrise to sunset. Once birds were collected, metrics were taken including fat and muscle scores, wing chord (mm), tarsometatarsus length (mm), and mass (g) (Table S1). Muscle score was determined by visually inspecting and feeling the pectoralis muscle using a 0–3 scale^29^. Fat score was determined by visually inspecting the furcular and abdominal cavity for subcutaneous fat using a 0–3 scale, modifying the 0–4 scale described by Salewski^29^. Our sample distribution included 33 females, 72 males, and 10 individuals of undetermined sex. Sex was determined by internal examination of the gonads. Males were determined by observing a testis during dissection whereas a female was determined by the presence of an ovary. The sex of the remaining individuals was not clear or missed. Capture tended to be episodic, so we held individuals for a maximum of 4 h in an enclosed screened 3 × 3 m tent with water and white proso millet provided ad libitum prior to humane euthanasia. We noted in our data whether an individual was held in the tent or immediately processed. Birds were sacrificed via decapitation in accordance with IACUC PRN #2019-3549 and following euthanasia techniques deemed humane based off the AVMA Guidelines for the Euthanasia of Animals: 2020 Edition^30^. The right pectoralis was immediately excised for mitochondrial respiration. Remaining pectoralis tissues not used for mitochondrial respiration were flash-frozen in liquid nitrogen and then later moved to a − 80 °C freezer for future analyses. For all plate assays, to control for variation between plates, the samples were equally divided by groups on 96 well plates randomly, thus the researcher could not be blind to sample identification. Since selection was random, there was not an equal number of samples per group per plate.

### Blood parameters and β-hydroxybutyrate

Blood was collected via venipuncture of the brachial vein prior to humane euthanasia using a 26-gauge needle. The blood was collected in 75 µl microhematocrit capillary tubes and did not exceed 1% of the bird’s body mass following standard procedures^31^. The blood was then centrifuged for 10 min at 17,700*g* to separate red blood cells from plasma. Hematocrit (% Hct) was measured as the percent erythrocytes over whole blood plasma. Hemoglobin (Hb) measures were taken by adding 5 µl of whole blood to 1.25 ml Drabkin’s solution. The solution was vortexed and then stored at 4 °C until future analysis. Hb levels were determined spectrophotometrically^32^. β-hydroxybutyrate (BOH) levels were determined using a ketone assay kit from Sigma-Aldrich (Product # MAK134). For % Hct, a minimum of two capillary tubes were measured and the mean was determined. Hb was measured in triplicate and BOH was measured in duplicate, and the mean was reported. For BOH, we removed the April data entirely because the intraassay CV was > 10% between replicates and we did not have enough plasma for additional re-runs for the April samples. We maintained inter-assay coefficient of variation (CV) of reported means ≤ 15% and intraassay ≤ 10%.

### Mitochondrial isolation and respiration

Mitochondria were isolated via differential centrifugation following procedures outlined previously^33^. An excised 1–2 g sample of the right pectoralis was quickly weighed and then put into a skeletal muscle isolation solution at a pH of 7.5 (100 mM KCl, 40 mM Tris–HCl, 10 mM Tris Base, 1 mM MgCl_2_, 1 mM EGTA, 0.2 mM ATP, and a 0.15% BSA solution) at a 1:10 ratio and then minced with scissors. The minced tissue was additionally homogenized for 5 s with a VITRIS electric homogenizer at half power. A protease from *Bacillus licheniformis* was made fresh using the isolation solution with BSA and added to the homogenate at 5 mg per gram of wet muscle for further digestion. The homogenate was mixed for 7 min by swirling vigorously every 30 s. Digestion was terminated by adding equal volume of original isolation solution. The homogenate was centrifuged at 500*g* for 10 min at 4 °C. The supernatant was decanted using cheese-cloth and centrifuged at 4500*g* for 15 min at 4 °C. This centrifuge step was repeated once after resuspending the pellet. The supernatant was again discarded and resuspended in isolation solution but without the 0.15% BSA solution. The pellet was centrifuged at 3500*g* for 10 min at 4 °C as an additional wash. Once the supernatant was removed, the final mitochondrial pellet was resuspended in ~ 0.25–0.75 ml of a mannitol-sucrose solution using rubber policeman. The sample was transferred to a Dounce homogenizer and resuspended with 4–5 passes.

We quantified mitochondrial respiratory states polarographically in a respiration chamber maintained at 40 °C (Oxytherm; Hansatech Instruments, United Kingdom)^34^. We used three substrates or substrate cocktails, pyruvate-malate-glutamate, palmitoyl-carnitine, and succinate, to induce mitochondrial respiration^35,36^. Ultimately, these substrates provide electrons to the ETS in the form of NADH (pyruvate-malate-glutamate, palmitoyl-carnitine) and FADH_2_ (succinate) and thus, test the capacity of complex I and complex II respiration. Because palmitoyl-carnitine must be processed via β-oxidation pathway before being converted to NADH in the citric acid cycle, measuring palmitoyl-carnitine also provides an indication of relative capacity for β-oxidation. This allows us to detect where differences in mitochondrial respiration performance occur between these subspecies.

Using these different substrates, we quantified mitochondrial states, which can be used to determine mitochondrial efficiency^37^. Because ATP is not measured, these values quantify oxygen utilization is a proxy for ATP production. State 3 is defined as the rate that ADP is converted to ATP when ADP is added with excess substrate and oxygen^33,37^. We refer to state 3 respiration as OXPHOS capacity, the maximum ATP production by coupled mitochondria^38^. Oligomycin was added to calculate oligomycin-induced state 4 (state 4o) and this is the state 4 we report in our results^39^. The addition of oligomycin prevents contamination of ATP-recycling molecules such as ATPases^40^. State 4 respiration is the minimum rate of oxygen utilization when substrate and oxygen are still abundant, but no ADP is present^37^. State 4 respiration is also a proxy for proton leak across the inner mitochondrial membrane, reducing respiration efficiency. Mitochondrial respiratory control is an indication of the relative performance of coupled mitochondria and is indicated by the respiratory control ratio (RCR), calculated by dividing state 3 by state 4 with oligomycin^33^.

These measurements were accomplished by adding a range of 5–30 μl of the isolated mitochondria incubated in respiration buffer (pH 7.0) with 220 mM Mannitol, 70 mM Sucrose, 10 mM Tris–HCl, and 1 mM EGTA at 40 °C. Respiration buffer amount was adjusted for total volume in the chamber to be 1 ml. Complex I respiration was tested using 2 mM pyruvate, 2 mM malate, and 10 mM glutamate (PMG) as substrates. We also tested complex I respiration using 4 mM of the substrate palmitoyl-carnitine (PC). Complex II was tested using 5 mM succinate (SUCC) with 10 μM rotenone to inhibit complex I. State 3 respiration was initiated by the addition of 0.25 mM ADP to the chambers containing the mitochondria and respiratory substrates. Respiration rates were normalized to total mitochondrial protein concentration using the Bradford assay technique.

### Citrate synthase and enzymatic activities

Citrate synthase activity assays were conducted as a proxy for mitochondrial volume of frozen pectoralis homogenate samples following methods previously outlined^20,41^. A volume of 750 µl of lysis buffer was added to 30–50 mg of tissue and homogenized and spun at 1500*g* for 15 min at 4 °C. The supernatant was collected, and protein content was determined using the Bradford assay technique. Citrate synthase activity was measured spectrographically at 40 °C as an increase in absorbance from 5,5′-dithiobis-2-nitrobenzoic acid reduction over a minute, with 10 s intervals. Spinazzi et al. was used to calculate the final CS values^42^.

Electron transport system complex activities I, III, and IV were determined using methodology from Spinazzi et al. with minor modifications^42^. Complex II activity was determined as previously described by Kavazis et al.^43^. All activities were determined spectrophometrically. Frozen isolated mitochondria samples were subjected to three freezing and thawing cycles to lyse membranes before analysis. Complex I (NADH: ubiquinone oxidoreductase) was measured as a function of the decrease in absorbance from NADH oxidation by decylubiquinone minus rotenone resistance activities. Complex II (succinate dehydrogenase) was measured as a function of the decrease in absorbance from 2,6-dichloroindophenol reduction. Complex III (decylubiquinol cytochrome *c * oxidoreductase) was measured as a function of the increase in absorbance from cytochrome *c* reduction minus antimycin A resistant activity. Complex IV (cytochrome *c * oxidase) was measured as a function of the decrease in absorbance from cytochrome *c* oxidation minus KCN-resistant activity. Complex activities were standardized to total protein content using the Bradford assay technique. Citrate synthase and complex activities were measured in triplicate with the mean reported. We maintained inter-assay coefficient of variation (CV) of reported means ≤ 15% and intraassay ≤ 10%.

### Data analyses

All statistical tests were completed using R version 4.2.3^44^ and RStudio version 2023.6.1.524 using the linear model function^45^. We treated groups in two different ways and reported results on both. First, we tested for significance between the two subspecies: Gambel’s versus Nuttall’s White-crowned Sparrows. Second, we tested for significance with all five groups separately treated as explanatory variables using linear regression in R. We used a Tukey’s honest significant difference test with the emmeans package to evaluate pairwise comparisons between all five groups to prevent a type I statistical error^46^. Dependent variables included state 3, state 4, and RCR for testing mitochondrial performance hypotheses. Other dependent variables included citrate synthase, complexes I–IV activities, hematocrit, hemoglobin, and BOH. The dependent variables were treated as continuous variables with a normal distribution of residuals. We also tested the assumptions of our linear models using methods outlined in Zuur et al.^47^. Descriptive statistics of all variables can be found in Supplementary Table S2.

Because we had both males and females and a few temporarily housed birds in our dataset, we first tested for potential sex and housing effects to determine how to appropriately conduct our analyses. While we present the summary below, refer to Supplementary Table S3 for detailed statistics. For sex, we tested for both overall sex effects and sex effects within the five groups for the pairwise comparisons. For housing, we tested the two housed groups separately. For complex activities and blood parameters, we detected no significant differences with treatment of sex (*p* > 0.05, Table S3). For respiration, we only found significance with state 3 and RCR using PMG as substrates where females were significantly higher overall versus males (F = 4.27, d.f. = 1, 94, State 3: β = − 89.3, p = 0.04; RCR; β = − 3.07, *p* = 0.04, Table S3). However, no difference was found when testing for this effect in all five groups separately (*p* > 0.05, Table S3). There were no significant differences with citrate synthase overall (F = 0.40, β = − 61.32, d.f. = 1, 102, *p* = 0.53, Table S3). Citrate synthase with Gambel’s females versus males during the fall timepoint was higher (F = 13.0, β = − 5.92, d.f. = 1, 17, *p* = 0.002; Fig. S2A) but when the females were removed (n = 6), it did not impact the overall results (Table S3). For BOH, we did find a significant difference between males and females overall where females had higher BOH than males although the F-statistic was rather small indicating little effect on the dependent variable (F = 4.69, β = − 1.68, d.f. = 1, 52, *p* = 0.04; Fig. S3). Because the effects were minimal, we removed the effect of sex from all results presented below.

Only a few individuals were temporarily housed during the September collection (Nuttall’s; n = 6, Gambel’s; n = 3) but we tested for differences between the housed versus unhoused individuals. No significant differences were found within Nuttall’s individuals with treatment of housing (*p* > 0.05, Table S3). For Gambel’s individuals, we found a significant difference with state 4 using the substrates PMG where the housed animals had higher values (F = 5.53, β = 17.8, d.f. = 1, 18, *p* = 0.03; Fig. S4A). However, when the housed Gambel’s individuals were removed (n = 3), it did not impact the overall pattern and significance of the results (Fig. S4B). The only other difference we observed was the unhoused Gambel’s individuals had significantly higher Hb than the housed (F = 8.20, β = − 2.03, d.f. = 1, 16, *p* = 0.01; Fig. S5A). Again, we removed the housed Gambel’s individuals (n = 3), and it did not impact the overall pattern of significance of results (Fig. S5B). We collapsed these data and removed the effect of temporary housing from the data below. Below we report state 4 values with oligomycin but see Supplemental Fig. S6 for results without which had none to only minor changes to the overall patterns observed.

## Results

The results of all statistical models are listed in Supplementary Table S3.

### Morphology and blood parameters

**Figure 1:**
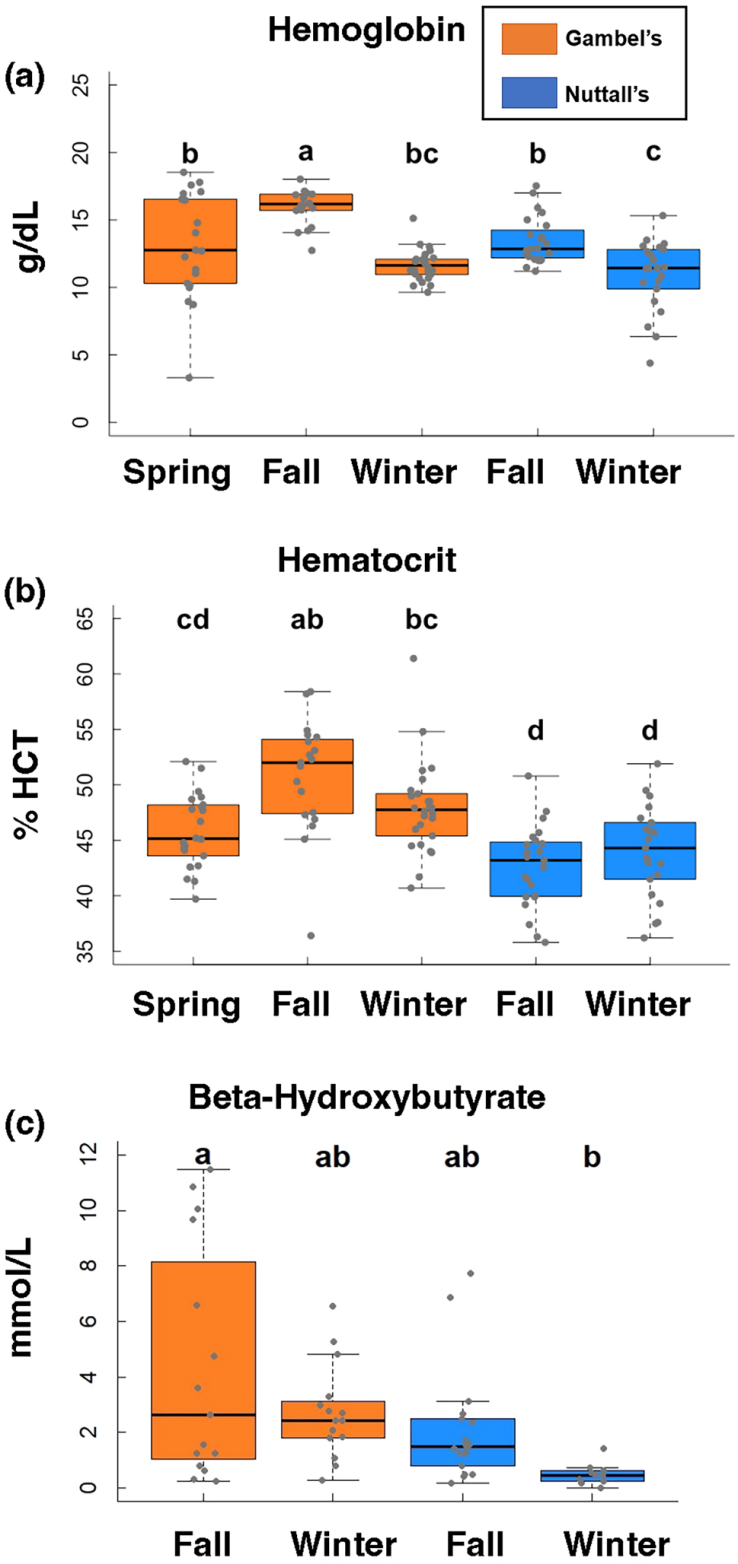
Blood parameters, ketones, and citrate synthase results. Hemoglobin (**a**), percent hematocrit (**b**), and β-hydroxybutyrate levels (**c**). The three Gambel’s group are represented as orange boxes and Nuttall’s as blue boxes. Significant differences are represented with letters. X-axes are the five collection timepoints. Y-axis is the variable of interest. The top and bottom of the boxes represent the upper (75%) and lower quartiles (25%). The median is represented as the black line in the middle of the boxes. The whiskers are the minimum and maximum values falling within the interquartile range times 1.5.

Gambel’s individuals had the highest fat and muscle scores recorded during the spring timepoint (Fig. S7). This was used to verify that these individuals were in a pre-migratory state. In comparison, we only observed fat (fat score > 0) in Nuttall’s individuals during the winter timepoint (Fig. S7). Overall, hemoglobin and hematocrit were significantly higher in Gambel’s than Nuttall’s individuals (Hb: F = 4.41, β = − 1.43, d.f. = 1, 108, *p* = 0.04, Fig. 1A) and (Hct: F = 32.2, β = − 4.78, d.f. = 1, 109, *p* < 0.001, Fig. 1B). Gambel’s individuals had higher Hb and % Hct in fall compared to the spring (Hb: Tukey, Z = − 3.65, β = − 2.75, d.f. = 105, *p* = 0.004; Hct: Tukey, Z = − 3.86, β = − 4.96, d.f. = 106, *p* = 0.002; Fig. 1A,B). Hb was lower in the winter than the spring for Gambel’s individuals but not significant (Tukey, Z = 2.31, β = 1.59, *p* = 0.15, Fig. 1A) and % Hct was similar for spring and winter (Tukey, Z = − 1.70, β = − 2.03, p = 0.44, Fig. 1B). For Nuttall’s individuals, Hb was significantly higher during the fall timepoint compared to winter (Tukey, Z = 3.58, β = 2.51, *p* = 0.005, Fig. 1A) but not for % Hct (Tukey, Z = − 1.04, β = − 1.28, *p* = 0.84, Fig. 1B). For between subspecies differences, BOH was significantly higher in Gambel’s than Nuttall’s individuals (F = 8.39, β = − 2.04, d.f. = 1, 56, *p* < 0.01, Fig. 1C). We did not find a significant difference between Gambel’s fall (mid-migration) and Gambel’s winter timepoints, however, the fall timepoint had a wider range of values than the winter (Tukey, Z = 1.72, β = 1.64, d.f. = 53, *p* = 0.36). There were no differences in BOH levels between the two Nuttall’s timepoints (Tukey, Z = 1.45, β = 1.55, *p* = 0.48, Fig. 1C).

### Citrate synthase

**Figure 2:**
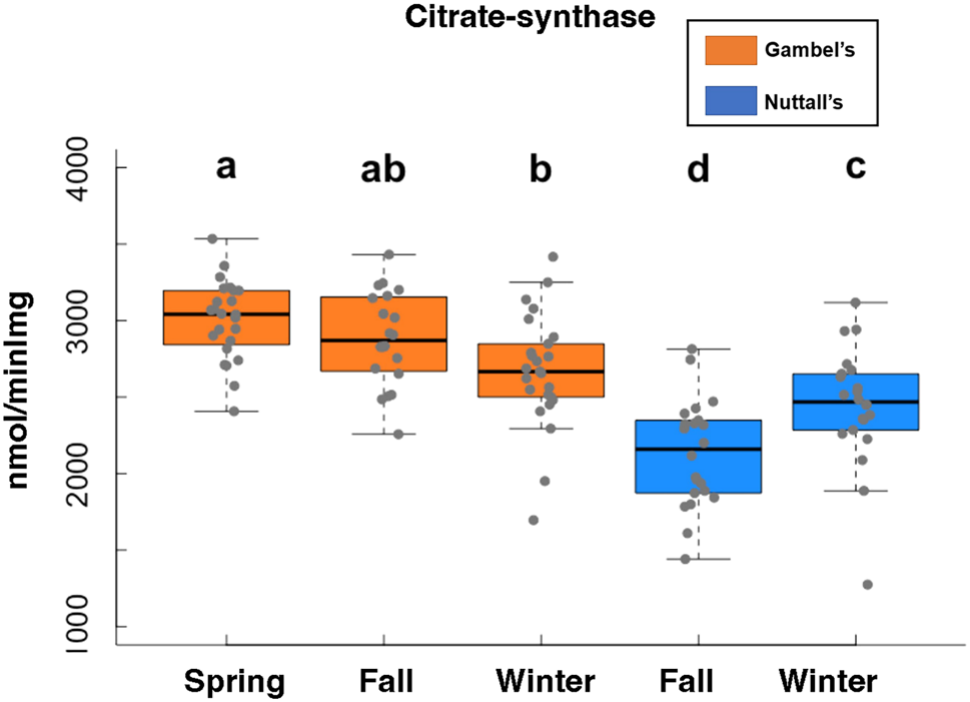
Citrate synthase results. Results of citrate synthase used as a proxy for mitochondrial volume. The Gambel’s are represented as orange boxes and Nuttall’s as blue boxes. Significant differences are represented with letters. X-axes are the five collection timepoints. Y-axis is the variable of interest. The top and bottom of the boxes represent the upper (75%) and lower quartiles (25%). The median is represented as the black line in the middle of the boxes. The whiskers are the minimum and maximum values falling within the interquartile range times 1.5.

Gambel’s individuals had higher citrate synthase compared to Nuttall’s individuals (F = 62.9, β = − 7.61, d.f. = 1, 111, *p* < 0.001, Fig. 2). Within Gambel’s individuals, the spring timepoint was significantly higher than Gambel’s winter timepoint (Tukey, Z = 3.46, β = 335, d.f. = 108, *p* = 0.007). However, the Gambel’s fall and spring nor fall and winter timepoints were not statistically different from one another (*p* > 0.05). All Gambel’s timepoints were significantly higher than Nuttall’s timepoints (*p* < 0.001) with exception to the winter Nuttall’s and winter Gambel’s timepoints (Tukey, Z = 2.27, β = 225, *p* = 0.16). There was a significant increase in citrate synthase observed within the Nuttall’s individuals for the winter versus fall timepoint (Tukey, Z = − 3.07, β = − 313, *p* = 0.02, Fig. 2).

### OXPHOS capacity (state 3)

**Figure 3:**
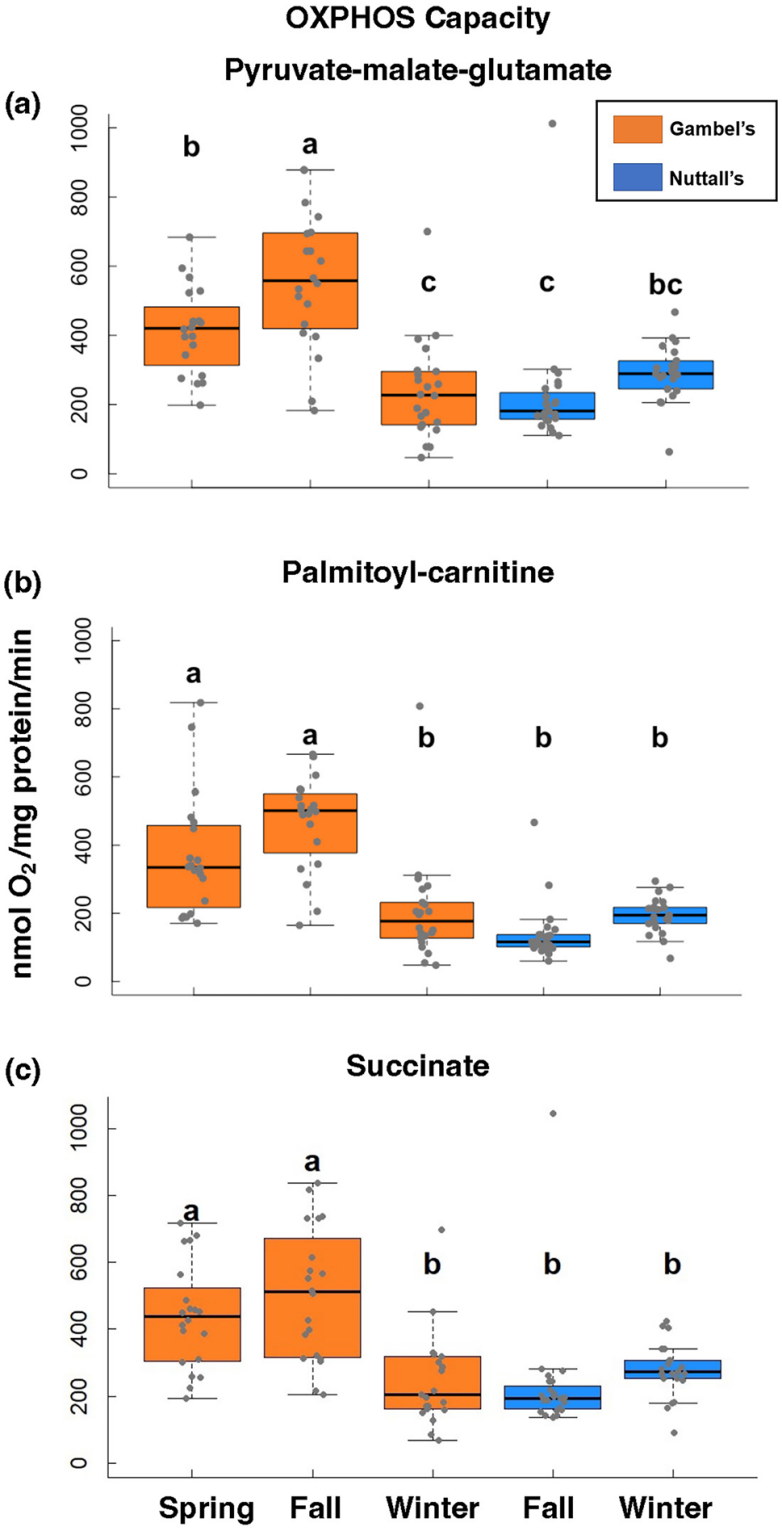
OXPHOS capacity results. Results of state 3 mitochondrial respiration using pyruvate-malate-glutamate (**a**), palmitoyl-carnitine (**b**), and succinate (**c**) as substrates. State 3 is defined as maximal rate of ADP to ATP conversion. The Gambel’s group are represented as orange boxes and Nuttall’s as blue boxes. Significant differences are represented with letters. X-axes are the five collection timepoints. Y-axis is the variable of interest. The top and bottom of the boxes represent the upper (75%) and lower quartiles (25%). The median is represented as the black line in the middle of the boxes. The whiskers are the minimum and maximum values falling within the interquartile range times 1.5.

Overall, OXPHOS capacity (state 3) was significantly higher for Gambel’s versus Nuttall’s individuals regardless of substrate (PMG: F = 15.5, β = − 141, d.f. = 1, 104, *p* < 0.001; PC: F = 33.9, β = − 176.13, d.f. = 1, 104, *p* < 0.001; SUCC: F = 15.8, β = − 138.86, d.f. = 1, 103, *p* < 0.001, Fig. 3). Based on pairwise comparisons, the Gambel’s fall timepoint had statistically higher OXPHOS capacity (state 3) compared to all other timepoints for complex I respiration using pyruvate-malate-glutamate (PMG) (d.f. = 101, *p* < 0.05, Fig. 3A) and palmitoyl-carnitine (PC) (d.f. = 101, *p* < 0.001, Fig. 3B) except it was not different than the Gambel’s spring timepoint using PC (Tukey, Z = − 2.40, β = − 97.9, *p* = 0.12). The two Nuttall’s timepoints were not significantly different from one another for complex I respiration using PMG (Tukey, Z = − 1.39, β = − 63.3, *p* = 0.63, Fig. 3A) or PC (Tukey, Z = − 1.43, β = − 55.5, *p* = 0.61, Fig. 3B). The Gambel’s fall timepoint was not statistically higher than the Gambel’s spring timepoint when using succinate (SUCC) (Tukey, Z = − 1.31, β = − 64.8, d.f. = 100, *p* = 0.68, Fig. 3C). Additionally using SUCC, Gambel’s individuals for both the spring and fall timepoint were higher than the winter timepoint for Gambel’s individuals (*p* < 0.01). The spring and fall timepoint for Gambel’s individuals using SUCC was also higher than Nuttall’s individuals in winter (Gambel’s Spring: d.f. = 100; Tukey, Z = 3.26, β = 159.90, *p* = 0.01; Fall: Tukey, Z = 4.58, β = 224.70, *p* < 0.01, Fig. 3C) and Nuttall’s individuals in fall (Gambel’s Spring: Tukey, Z = 4.26, β = 204, *p* < 0.001; Fall: Tukey, Z = 5.61, β = 269, *p* < 0.001, Fig. 3C). The winter Gambel’s timepoint was not statistically different than either of the Nuttall’s timepoints using all three substrates/substate cocktails (Gambel’s Winter: PMG: Tukey, Z = − 1.14, β = − 52.2, *p* = 0.788; PC: Tukey, Z = 0.25, β = 9.84, *p* = 1.0; SUCC: Tukey, Z = − 0.631 , β = − 30.6, *p* = 0.97; Fall: PMG: Tukey, Z = 0.247, β = 11.1, *p* = 1.0; PC: Tukey, Z = 1.70, β = 65.3, *p* = 0.439; SUCC: Tukey, Z = 0.30, β = 14.0, *p* = 1). Lastly, no significant differences were observed for SUCC for the two Nuttall’s timepoints (Tukey, Z = − 0.94, β = − 44.6, *p* = 0.88, Fig. 3C).

### Basal respiration (state 4)

**Figure 4:**
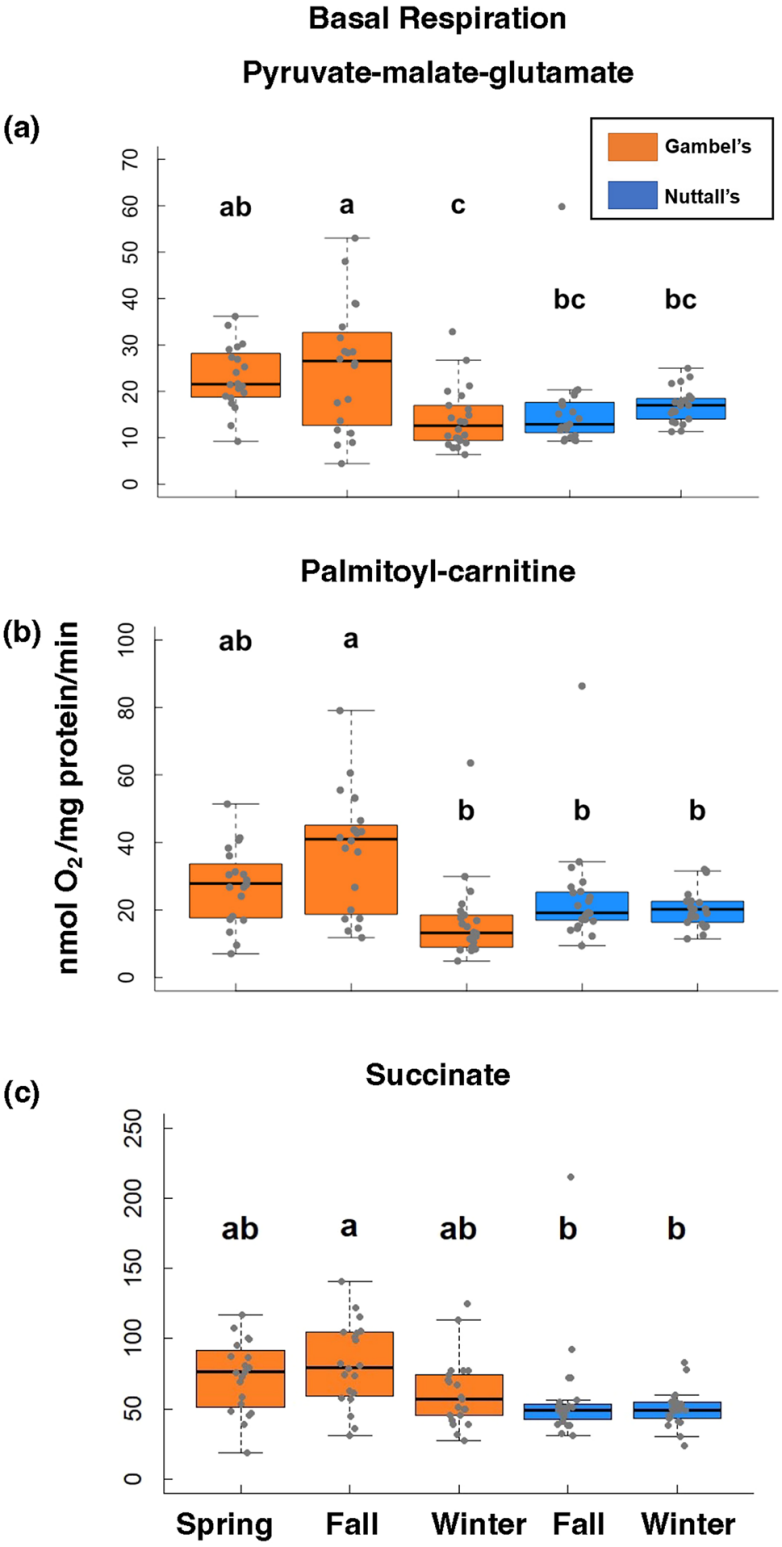
Basal (state 4) mitochondrial respiration results. Results of basal (state 4) mitochondrial respiration using pyruvate-malate-glutamate (**a**), palmitoyl-carnitine (**b**), and succinate (**c**) as substrates. Basal respiration (state 4) was determined after state 3 slowed and defined as minimum amount of oxygen consumption to maintain proton-motive force. State 4 is defined using oligomycin. The Gambel’s group are represented as orange boxes and Nuttall’s as blue boxes. Significant differences are represented with letters. X-axes are the five collection timepoints. Y-axis is the variable of interest. The top and bottom of the boxes represent the upper (75%) and lower quartiles (25%). The median is represented as the black line in the middle of the boxes. The whiskers are the minimum and maximum values falling within the interquartile range times 1.5.

Basal respiration (state 4) was also significantly higher for Gambel’s versus Nuttall’s individuals for PMG (F = 4.73, β = − 4.06, d.f. = 1, 104, *p* = 0.03, Fig. 4A) and SUCC (F = 10.5, β = − 17.84, d.f. = 1, 103, *p* = 0.002, Fig. 4C) but not PC (F = 2.82, β = − 4.80, d.f. = 1, 104, *p* = 0.10, Fig. 4B). Based on pairwise comparisons, basal respiration (state 4) was significantly higher for Gambel’s individuals in the fall versus winter timepoint for complex I using PMG (Tukey, Z = 4.04, β = 11.0, d.f. = 101, *p* = 0.001, Fig. 4A) as well as the fall Nuttall’s timepoint (Tukey, Z = 3.38, β = 9.1, *p* = 0.009) and winter Nuttall’s timepoint (Tukey, Z = 2.94, β = 8.10, *p* = 0.03). However, Gambel’s fall versus spring timepoints were not statistically different (Tukey, Z = − 0.75, β = − 2.08, *p* = 0.95). For complex I using PC, state 4 for the Gambel’s fall timepoint was significantly higher than the winter Gambel’s timepoint (Tukey, Z = 5.16, β = 20.8, d.f. = 101, *p* < 0.001, Fig. 4B), the fall Nuttall’s timepoint (Tukey, Z = 3.52, β = 14.04, *p* = 0.006), and winter Nuttall’s timepoint (Tukey, Z = 4.16, β = 17.00, *p* < 0.001). Gambel’s fall was not different then the Gambel’s spring timepoint (Tukey, Z = − 2.45, β = − 10.12, *p* = 0.11, Fig. 4B). For complex II using SUCC, the Gambel’s fall timepoint was significantly higher than the fall Nuttall’s timepoint (Tukey, Z = 2.89, β = 24.2, d.f. = 100, *p* = 0.04, Fig. 4C) and winter Nuttall’s timepoint (Tukey, Z = 3.71, β = 31.69, *p* = 0.003). However, the Gambel’s fall and spring timepoints were not statistically different (Tukey, Z = − 1.01, β = − 8.77, *p* = 0.85, Fig. 4C) as well as the winter Gambel’s timepoint (Tukey, Z = 2.39, β = 20.4, *p* = 0.13). The Nuttall’s timepoints were not significantly different for complex I using PMG (Tukey, Z = − 0.38, β = − 1.01, d.f. = 101, *p* = 1, Fig. 4A), PC (Tukey, Z = 0.75, β = 2.95, d.f. = 101, *p* = 0.94, Fig. 4B), and SUCC (Tukey, Z = 0.91, β = 7.54, d.f. = 100, *p* = 0.89, Fig. 4C).

### Mitochondrial respiratory capacity (RCR)

**Figure 5:**
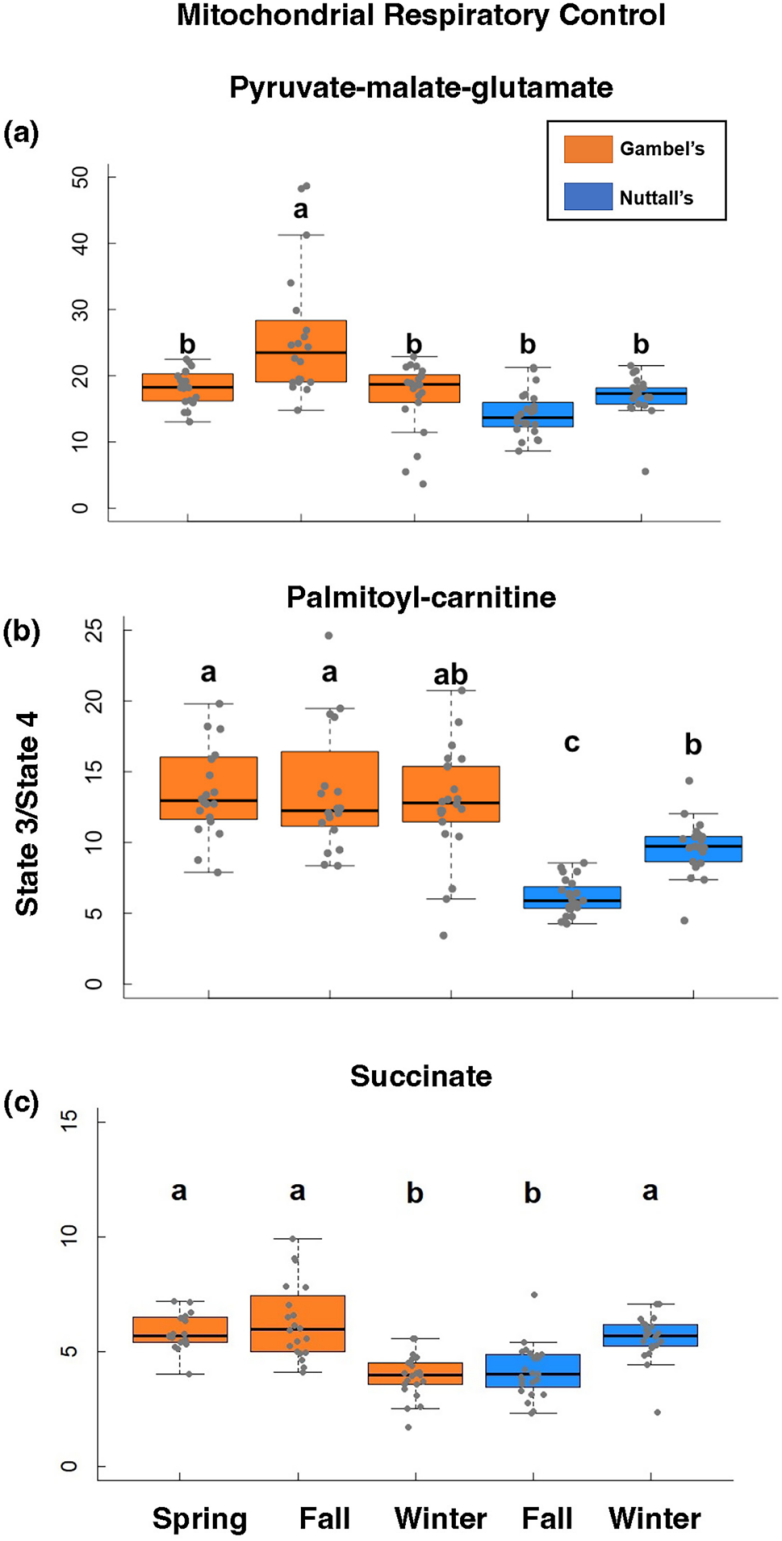
Mitochondrial respiratory control results. results of RCR (Respiratory Control Ratio or state 3/state 4) using pyruvate-malate-glutamate (**a**), palmitoyl-carnitine (**b**), and succinate (**c**) as substrates. RCR is the range at which mitochondria perform. The Gambel’s group are represented as orange boxes and Nuttall’s as blue boxes. Significant differences are represented with letters. X-axes are the five collection timepoints. Y-axis is the variable of interest. The top and bottom of the boxes represent the upper (75%) and lower quartiles (25%). The median is represented as the black line in the middle of the boxes. The whiskers are the minimum and maximum values falling within the interquartile range times 1.5.

RCR was significantly higher for Gambel’s versus Nuttall’s individuals for PMG (F = 14.9, β = − 4.76, d.f. = 1, 104, *p* < 0.001, Fig. 5A) and PC (F = 55.5, β = − 5.90, d.f. = 1, 104, *p* < 0.001, Fig. 5B), with exception of SUCC (F = 3.44, β = − 0.67, d.f. = 1, 103, *p* = 0.07, Fig. 5C). Based on pairwise comparisons, fall was significantly higher than the spring and winter timepoint for the Gambel’s timepoints with PMG (Spring: d.f. = 101, Tukey, Z = − 4.52, β = − 7.80, *p* < 0.01; Winter: Tukey, Z = 5.37, β = 9.06, *p* = < 0.001 Fig. 5A); however, the spring and winter timepoints were not significantly different (Tukey, Z = 0.75, β = 1.26, *p* = 0.94). While the fall timepoint for Gambel’s individuals was higher than both Nuttall’s timepoints (*p* < 0.001), there were no differences between the winter Gambel’s and both Nuttall’s timepoints (*p* > 0.05). The spring timepoint for Gambel’s individuals was not significantly different than the fall or winter Nuttall’s individuals (*p* > 0.05). The two Nuttall’s timepoints for complex I using PMG were not significantly different (Tukey, Z = − 1.70, β = − 2.80, *p* = 0.44, Fig. 5A). All timepoints for Gambel’s were significantly higher than the Nuttall’s for complex I using PC (d.f. = 101, *p* < 0.01, Fig. 5B) with exception to Gambel’s winter versus Nuttall’s winter (Tukey, Z = 2.65, β = 3.13, *p* = 0.07, Fig. 5B). There were no significant differences between the three Gambel’s timepoints (*p* > 0.05, Fig. 5B). The winter timepoint for Nuttall’s individuals was higher compared to the fall Nuttall’s timepoint for complex I using PC (Tukey, Z = − 3.04, β = − 3.55, *p* = 0.02, Fig. 5B). For SUCC comparing Gambel’s individuals, the spring and fall timepoints were higher than the winter timepoint (d.f. = 100, *p* < 0.001, Fig. 5B) although there were no significant differences between the Gambel’s spring and fall timepoints (Tukey, Z = 0.10, β = 0.05, *p* = 1). The winter Nuttall’s timepoint was significantly higher than the Gambel’s winter timepoint using SUCC (Tukey, Z = − 3.53, β = − 1.67, *p* = 0.006, Fig. 5C). The winter timepoint for Nuttall’s individuals was higher compared to the fall Nuttall’s timepoint for complex II using SUCC (Tukey, Z = − 3.16, β = − 1.47, *p* = 0.02, Fig. 5C).

### Complex enzymatic activity

**Figure 6:**
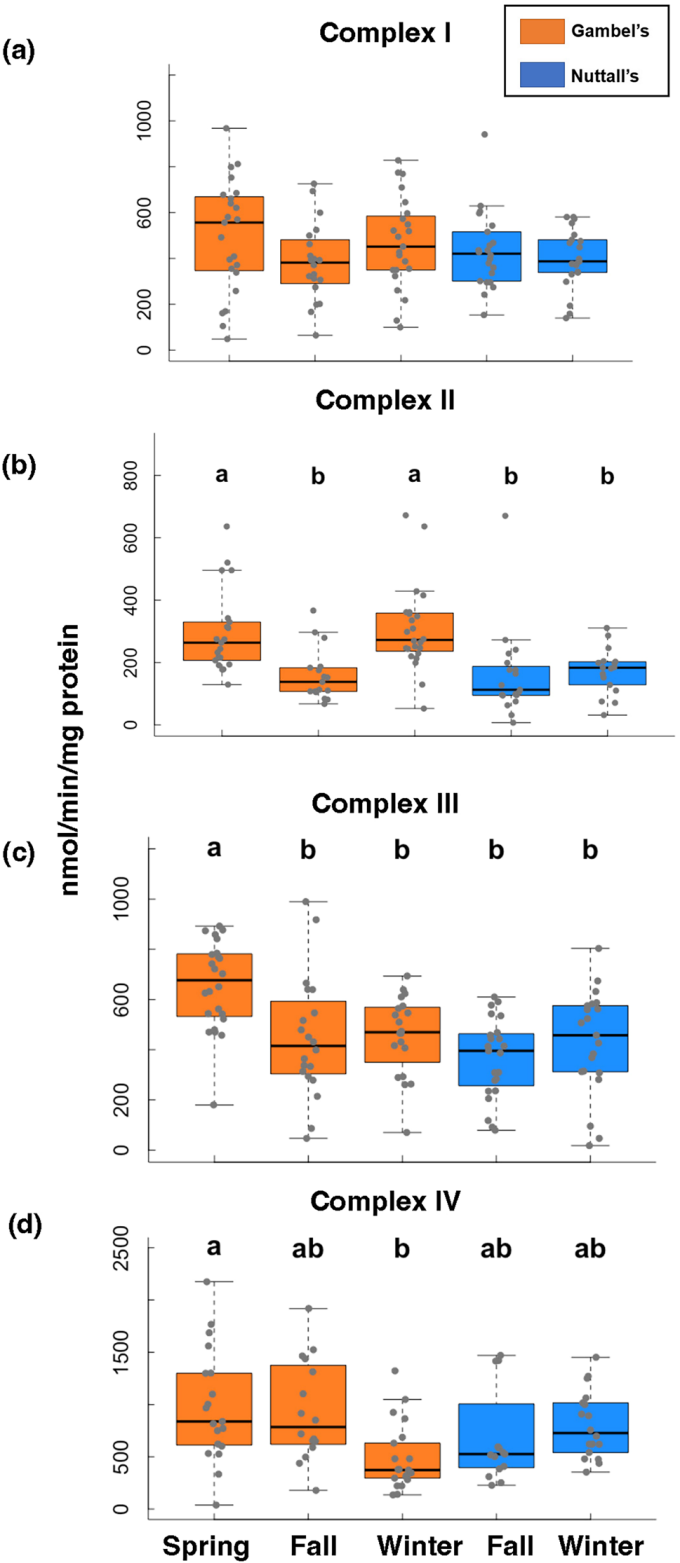
Mitochondrial complex enzymatic activities (I–IV) results. All activities were determined spectrophometrically. The Gambel’s are represented as orange boxes and Nuttall’s as blue boxes. Significant differences are represented with letters. X-axes are the five collection timepoints. Y-axis is the variable of interest. The top and bottom of the boxes represent the upper (75%) and lower quartiles (25%). The median is represented as the black line in the middle of the boxes. The whiskers are the minimum and maximum values falling within the interquartile range times 1.5.

For complex activity, complex I and IV were not significantly between Gambel’s or Nuttall’s individuals (CI: F = 0.94, β = − 36.01, d.f. = 1, 107, *p* = 0.34; CIV: F = 0.10, β = 30.20, d.f. = 1, 87, *p* = 0.75, Fig. 6A,D). For complex II and III, Gambel’s individuals were significantly higher than Nuttall’s individuals (CII: F = 12.96, β = − 97.49, d.f. = 1, 96, *p* <0.001, CIII: F = 11.03, β = − 132.02, d.f. = 1, 106, *p* = 0.001, Fig. 6C). Based on pairwise comparisons, there were no differences with any of the pairwise comparisons for complex I (d.f. = 104, *p* > 0.05, Fig. 6A). For complex II, Gambel’s fall timepoint was significantly lower than winter (Tukey, Z = − 3.86, β = − 147, d.f. = 93, *p* = < 0.01, Fig. 6B) and spring timepoints (Tukey, Z = 3.51, β = 138, *p* = < 0.01, Fig. 6B). For complex III, the only significant difference was the Gambel’s spring timepoint was statistically higher than all the other timepoints (d.f. = 103, *p* < 0.01, Fig. 6C). For complex IV, the Gambel’s spring was significantly higher than the Gambel’s winter (Tukey, Z = 3.07, β = 499, d.f. = 84, *p* = 0.03, Fig. 6D). No significant differences were detected between the Nuttall’s for all complexes (*p* > 0.05, Fig. 6A–D).

## Discussion

While numerous studies have suggested that migratory species support higher energetic demands than individuals that are non-migratory^3,48,49^, few studies have explored mitochondrial respiratory performance in the organs that generate this energy demand. For this study, we examined mitochondrial respiratory performance in a migratory and non-migratory subspecies of White-crowned Sparrows. We observed higher OXPHOS capacity in migratory Gambel’s relative to non-migratory Nuttall’s individuals for all mitochondrial substrates. Further, we found that the Gambel’s individuals displayed flexibility in the performance of ETS for all substrates, while Nuttall’s individuals displayed limited and low magnitude flexibility in the winter, which is likely a thermogenic response. Our results support the OXPHOS flexibility hypothesis that the bioenergetic underpinnings of migration are flexible within individuals in White-crowned Sparrows.

We found that hematocrit, hemoglobin, and β-hydroxybutyrate were all higher in Gambel’s than Nuttall’s sparrows, supporting the assumption that oxygen and substrate delivery to the pectoralis was higher in the migratory population. We also observed that citrate synthase was higher in Gambel’s than Nuttall’s individuals, indicating that the Gambel’s individuals likely have greater mitochondrial volume that Nuttall’s individuals. The Gambel’s population displayed phenotypic flexibility across the evaluated timepoints, but the patterns were not identical for all variables. Hematocrit and hemoglobin were elevated in Gambel’s individuals only during period of active migration, suggesting that variables that increase oxygen carrying capacity respond to the demands of flight rather than being upregulated in anticipation of migration (but see Krause et al.^13^). A similar increase in oxygen carrying capacity in response to energy demands is observed in humans training at altitude^50^. Similarly, β-hydroxybutyrate was also elevated only during migration, indicating that the availability of ketone bodies was high during migration in Gambel’s individuals but low during the non-migratory period. Frias-Soler et al. showed that 13 different genes regulating ketogenesis are differentially expressed relative to migratory status in the migratory Northern Wheatear (*Oenanthe oenanthe*)^26^ providing added support for the hypothesis that ketone bodies are vital for pre-migratory fattening and long-distance migratory flight.

In contrast to the changes in hematocrit, hemoglobin, and β-hydroxybutyrate that appear to be in response to energy demands of migration, citrate synthase appears to be up-regulated in preparation for migration; data indicates that Gambel’s individuals have a higher volume of mitochondria in the pectoralis prior to as well as during migration compared to volume during winter, although the differences were small. Interestingly, Price et al. could not induce changes in citrate synthase with a shift to a migratory photoperiod or with exercise in the lab in Gambel’s White-crowned Sparrows^21^. Western Sandpiper (*Calidris mauri*) females display higher citrate synthase during migration, although the pre-migratory timepoint was not as high as migration in this study nor was it different from the winter non-migratory timepoint. Additionally, higher citrate synthase was not found in males^17^. These findings suggest that, in contrast to oxygen delivery, Gambel’s sparrows appear to upregulate mitochondrial volume before they begin migratory flights. While the drop in citrate synthase was limited during winter in Gambel’s individuals, we observed an increase in citrate synthase in Nuttall’s during the winter timepoint compared to fall. Previous work has attributed relatively high citrate synthase values to increased thermogenic capacity^51^, and Nuttall’s sparrows are known to have an increase in energetic demands during the winter correlated with increased precipitation and decreased temperatures^52^. It is also possible that keeping citrate synthase relatively high in Gambel’s individuals also supports thermogenesis.

We evaluated OXPHOS capacity of pectoralis mitochondria using three different substrates/substrate cocktails, which evaluate the performance of the ETS when electrons are provided to the ETS via complex I (PMG, PC) or complex II (SUCC). As predicted by symmorphosis, mitochondrial respiratory performance was higher prior to and during migration, suggesting that adaptations that improve oxygen and nutrient supply to the cells, to the supporting pathways of β-oxidation, and to the TCA cycle are closely linked to the respiratory demands of OXPHOS. Interestingly, all substrates yielded nearly the same results, with Gambel’s and Nuttall’s individuals being statistically different from one another. The subspecies-specific differences in OXPHOS capacity that we observed between subspecies of White-crowned Sparrows are consistent with observations for migratory and non-migratory Yellow-rumped Warblers as indicated by another measure of oxidative coupling, the acceptor control ratio^22^. Our results demonstrated that OXPHOS capacity (state 3) in Gambel’s individuals was higher during the spring pre-migratory and migratory periods relative to the winter non-migratory period for all substrates, although not significantly so for complex II respiration. This suggests that it is likely that individuals display an upregulation of OXPHOS capacity in preparation for migration.

OXPHOS capacity was numerically highest during active migration for all substrates, although it was statistically different only for complex I respiration with PMG. The OXPHOS capacity of winter Gambel’s individuals was not different than either of the Nuttall’s individuals timepoints. Higher OXPHOS capacity during the migratory period would provide the pectoralis muscle with the potential to generate more ATP than it could at the other timepoints. Further, although not compared directly, OXPHOS capacity values for all substrates are relatively similar suggesting that, when substrates are in excess, there is little difference in the ability of the ETS to run OXPHOS via complex I, whether running through β-oxidation first or not, or complex II using succinate. Basal respiration (state 4), a proxy for proton leak, also displayed similar patterns, suggesting that proton leak increased with OXPHOS capacity and was low outside of the migratory period and in non-migrants.

Mitochondrial respiratory capacity (RCR) not only provides a measure of coupling efficiency; it also gives some perspective on the relationship between state 3 and state 4 respiration^37^. The patterns observed for changes in mitochondrial respiration for White-crowned Sparrows were not consistent between substrates, nor did they consistently follow the pattern observed for OXPHOS capacity. For all comparisons, mitochondrial respiratory capacity was greater for Gambel’s than Nuttall’s sparrows suggesting that Gambel’s individuals have a greater coupling efficiency, with the implication being that leak makes a smaller contribution to OXPHOS capacity for individuals from the migratory versus non-migratory populations. The similarity in RCR when using palmitoyl-carnitine which runs through the β-oxidation pathway and the citric acid cycle prior to OXPHOS, suggest Gambel’s individuals have a high capacity for utilizing fatty acids to support OXPHOS across all timepoints, with protons lost to leak making up a relatively similar proportion of OXPHOS across all timepoints. This aligns with previous work demonstrating an upregulation of carnitine palmitoyl-transferase in pectoralis during active migration^17,21^.

Finally, we measured the enzymatic activity of complexes of the ETS to investigate the mechanisms responsible for the patterns of mitochondrial respiration that we observed. While caution must be taken when interpretating complex activity results, because it may not represent true mitochondrial functionality^37^, complex activities have the potential to reveal mechanisms responsible for the observed patterns of mitochondrial respiration that are otherwise masked^53^. While we found small differences between the Gambel’s timepoints and no differences between the Nuttall’s timepoints, it is important to note that none of the complexes displayed a pattern of oxygen utilization that mimics the patterns of OXPHOS observed for all mitochondrial substrates. This suggests that no individual complex played a major role in determining the patterns observed.

Interestingly, we observed lower complex II activity for mid-migration in Gambel’s individuals. One possible explanation could be that this reduction counters superoxide production. While superoxide production by complex II in healthy mammals is negligible^54^, complex II has been shown to be a source of superoxide production when oxidizing palmitoyl-carnitine^55–57^. Nevertheless, because complex II respiration was elevated for all substrates at this time point, we are cautious to suggest that this is a biologically relevant finding. It is feasible that the upregulation in complex III prior to migration plays some role in imitating the increase in OXPHOS for migration, but it is odd that this is not also found during migration. Further, there is no obvious explanation for lower complex IV activity in Gambel’s during winter. Future work on complex activity in migrants is warranted to see if these patterns persist in other species.

**Figure 7:**
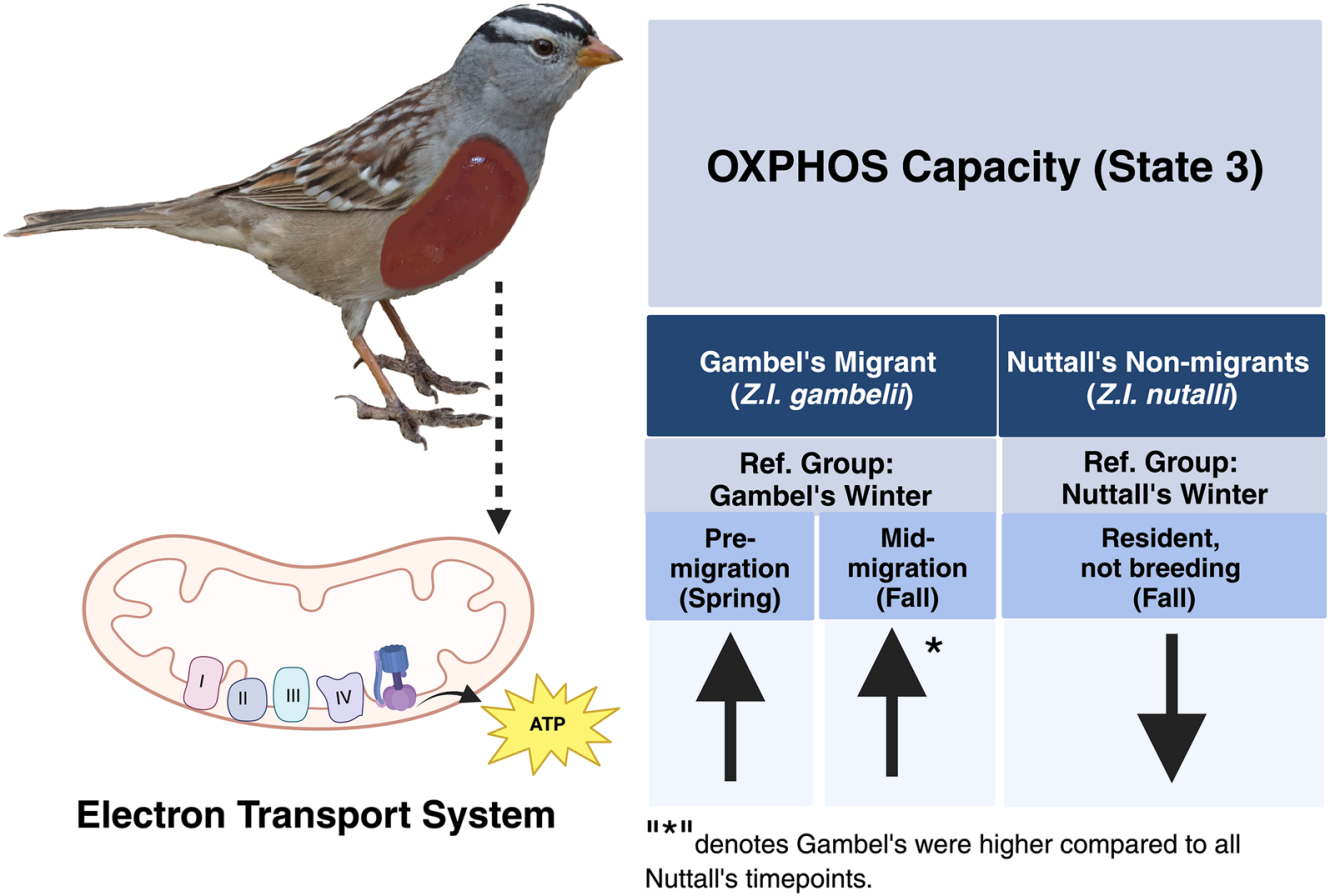
Summary figure of patterns observed with OXPHOS capacity (state 3) in support of the OXPHOS flexibility hypothesis. Within Gambel’s, the up arrows represent significantly higher values for state 3 for both the pre-migratory (spring) and mid-migration (fall) timepoints when compared to the winter timepoint. For Nuttall’s, the fall timepoint (resident, not breeding) for state 3 was lower when compared to the winter timepoint. The asterisk denotes that the mid-migration (fall timepoint) for Gambel’s was always higher than the Nuttall’s timepoints. Created with BioRender.com.

In summary, we observed that Gambel’s White-crowned Sparrows appear to adapt to the bioenergetic challenge of migration largely using phenotypic flexibility (Fig. 7). In particular, we observed that flexibility in mitochondrial respiratory performance, in addition to mitochondrial volume, are likely vital to supporting the energy demands of long-distance migration in vivo. A major drawback of conducting studies with isolated mitochondria is that the cellular control of substrate availability and the vivo morphology of the mitochondria lost are^37,58^, both of which are likely important to supporting migratory movements. Therefore, we cannot say for certain that the patterns of mitochondrial respiratory performance that we describe here precisely mimic patterns that occur in vivo. Recent studies have demonstrated the need for studying mitochondria in an evolutionary and ecological context^38^, and here we provide evidence that mitochondria play an important role in migration energetics. Future work should focus on the mechanisms controlling the upregulation of mitochondrial respiratory performance and how it correlates with other variables related to migration.

### Supplementary Information

Supplementary Figures.

Supplementary Table S1.

Supplementary Table S2.

Supplementary Table S3.

## Data Availability

All data generated or analyzed during this study are included in this published article (and its Supplementary Information files).
