# Supplementary material for: Flexibility underlies differences in mitochondrial respiratory performance between migratory and non-migratory White-crowned Sparrows (Zonotrichia leucophrys)
Source: Sci Rep. 2024 Apr 24;14:9456. doi: 10.1038/s41598-024-59715-y (PMC11043447; doi:10.1038/s41598-024-59715-y)
Supplement: Supplementary file 1 — Supplementary Figures. [file 41598_2024_59715_MOESM1_ESM.pdf]

Supporting information

Flexibility underlies differences in mitochondrial respiratory performance between migratory and non-migratory White-crowned Sparrows (*Zonotrichia leucophrys*)

Emma M. Rhodes[1], Kang Nian Yap[1,2], Paulo H. C. Mesquita[3,6], Hailey A. Parry[3,4], Andreas N. Kavazis[3], Jesse S. Krause[5], Geoffrey E. Hill[1], Wendy R. Hood[1]

[1]Department of Biological Sciences, Auburn University, Auburn, USA
[2]Department of Biology, Norwegian University of Science and Technology, Trondheim, Norway
[3]School of Kinesiology, Auburn University, Auburn, USA
[4]National Heart, Lung and Blood Institute, National Institutes of Health, Bethesda, USA
[5]Department of Biology, University of Nevada, Reno, USA
[6]Aging and Metabolism Research Program, Oklahoma Medical Research Foundation, Oklahoma City, USA

Corresponding author
Emma M. Rhodes
Email: emr0063@auburn.edu

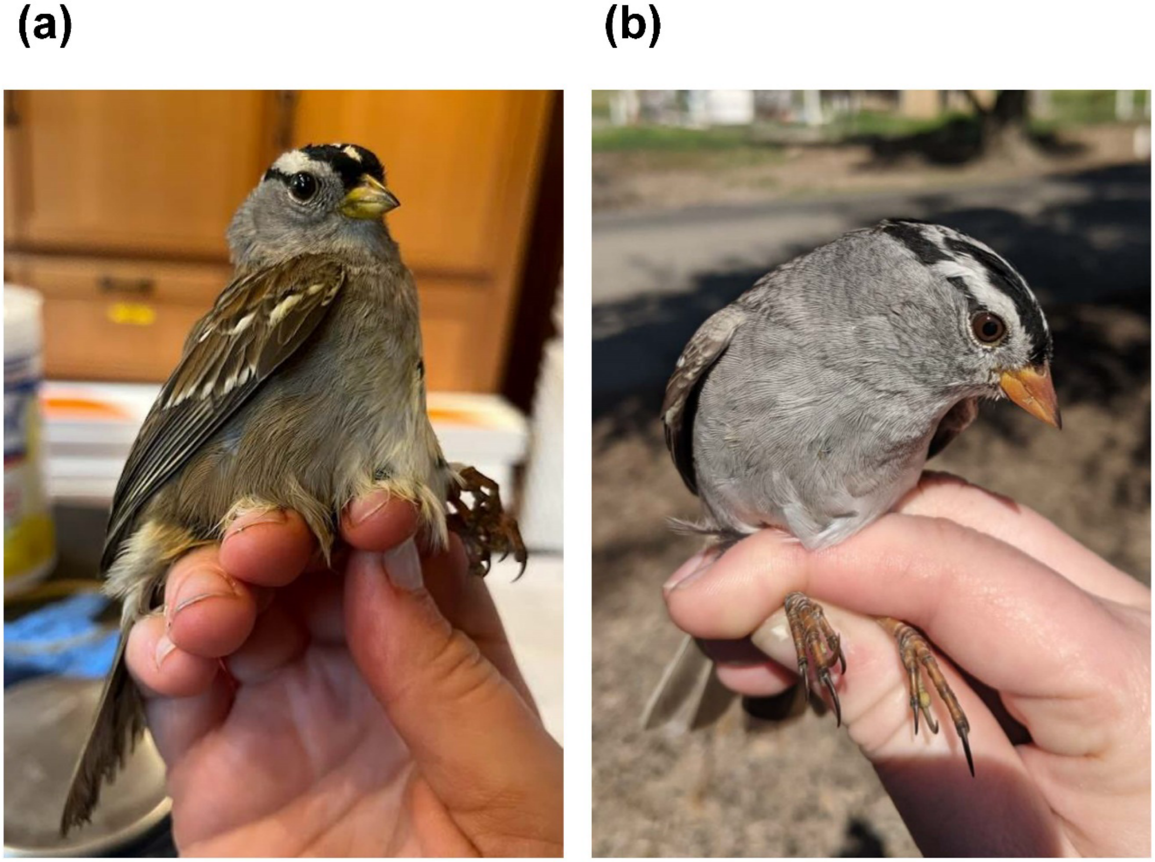

Figure S1. The two study groups. (a) Nuttall's and Gambel's White-crowned Sparrows (b).

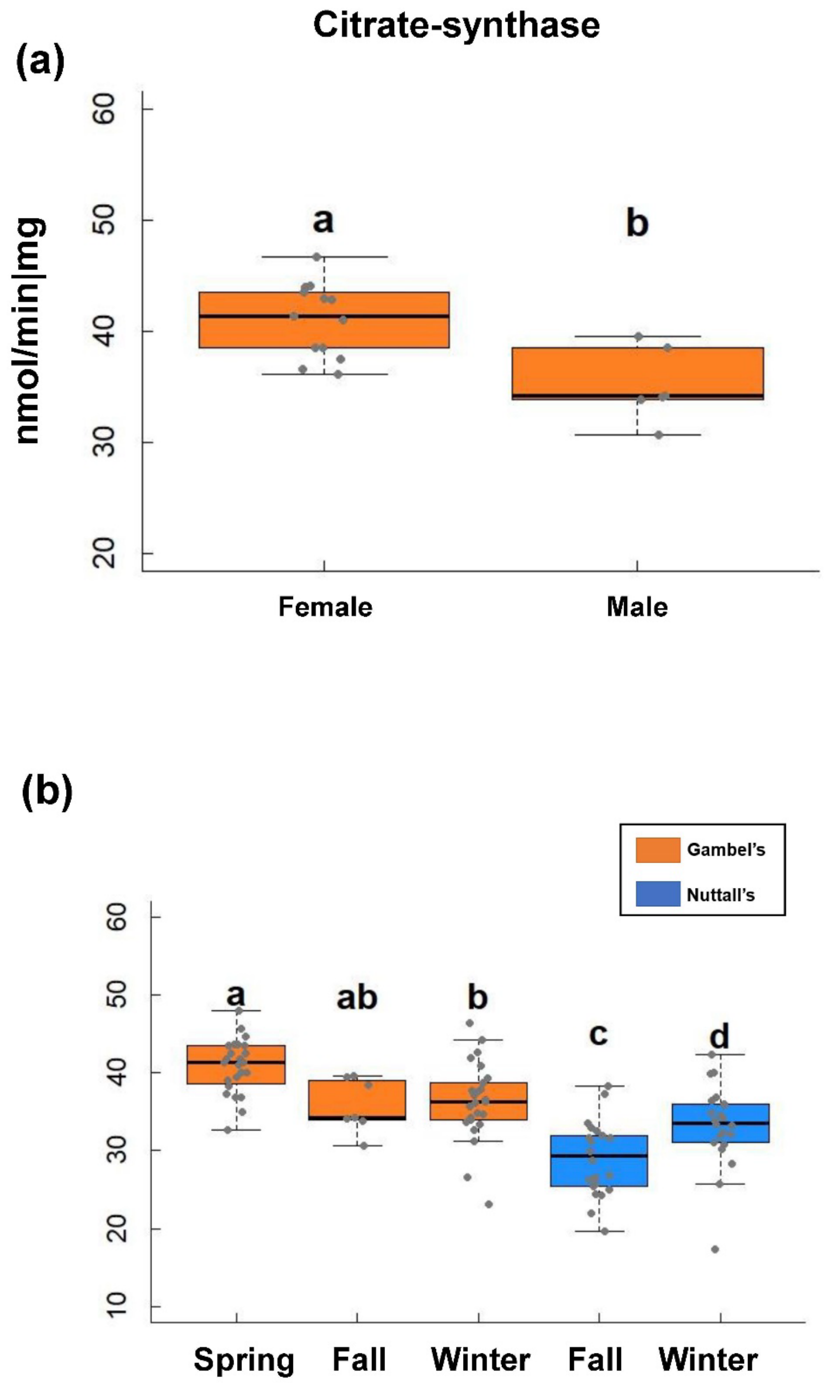

Figure S2. Effect of sex on Citrate-synthase. Results for effect of sex on citrate-synthase (a) and overall citrate-synthase results with females removed (b). Significant differences are represented with letters.

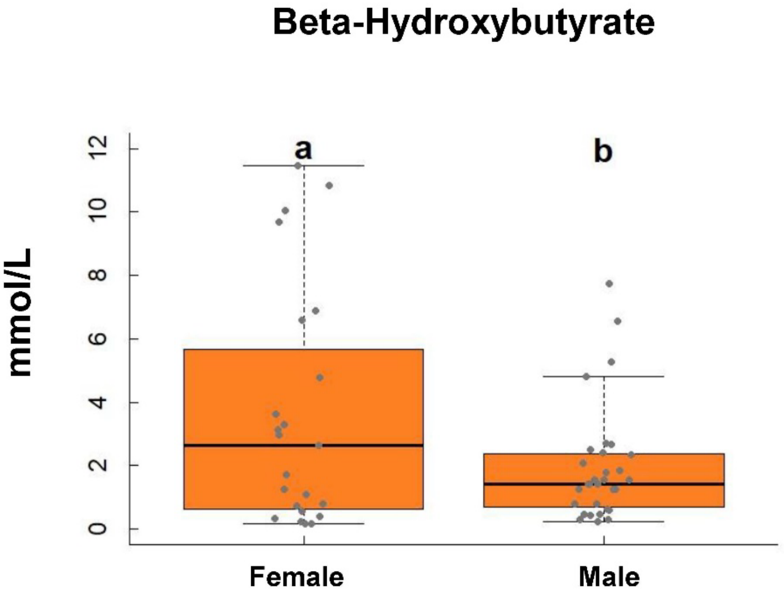

Figure S3. Results for effect of sex on ketone Beta-Hydroxybutyrate (BOH). Significant differences are represented with letters.

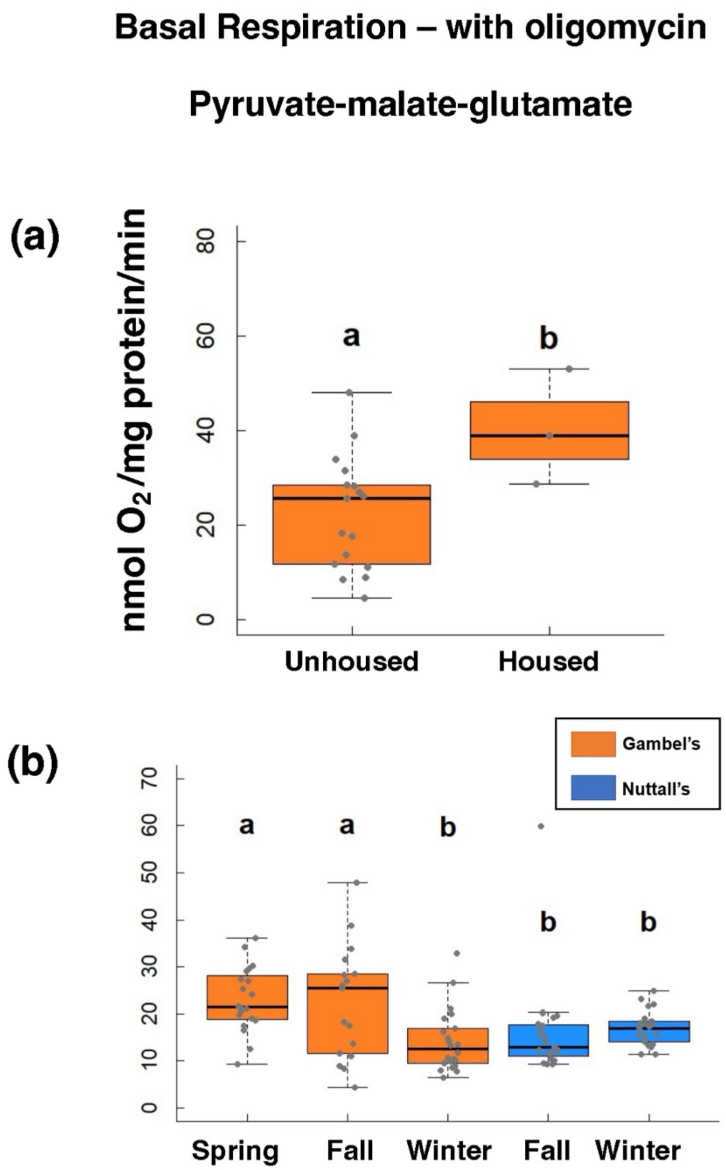

Figure S4. Effect of housing on State 4 PMG Respiration. Results for State 4 respiration using PMG substrates looking at the effect of temporarily housed (≤ 4HR) individuals versus unhoused (a). Overall results with individuals housed removed (b). Significant differences are represented with letters.

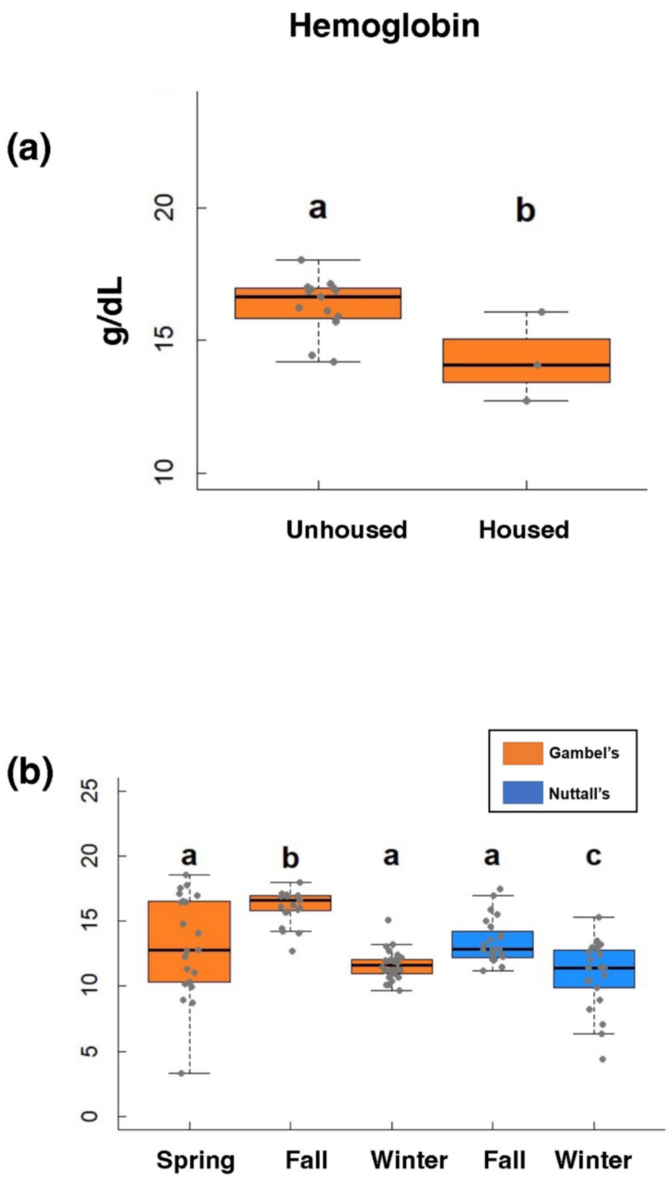

Figure S5. Effect of housing on Hemoglobin (Hb). Results for hemoglobin (Hb) looking at the effect of temporarily housed (≤ 4HR) individuals versus unhoused (a). Overall results with individuals housed removed (b). Significant differences are represented with letters.

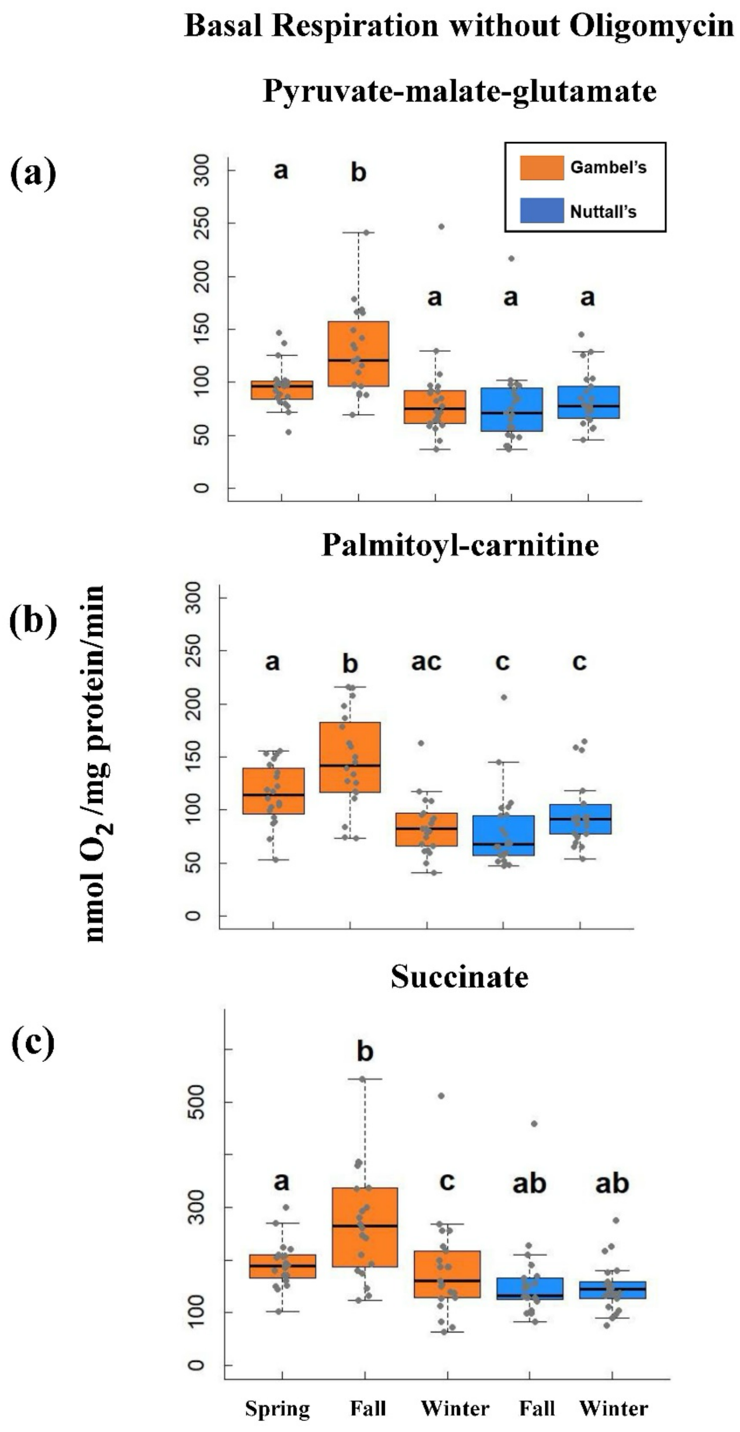

Figure S6. State 4 results without oligomycin. Results for State 4 respiration without oligomycin using all three substrates with all groups.

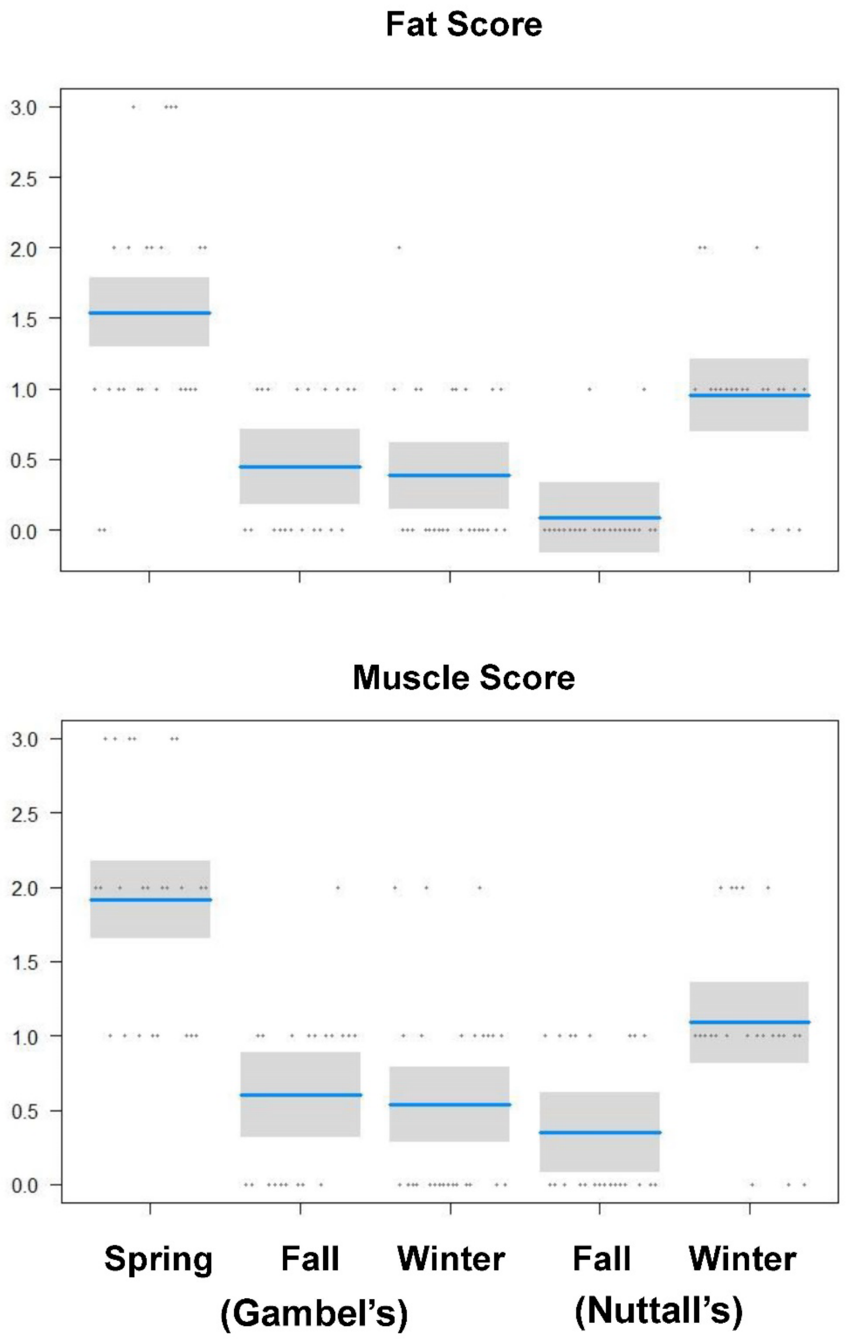

Figure S7. Fat and muscle scores by group. Fat and muscle scores using a 0-3 categorization.
